# Incidence of Epilepsy and Seizures Over the First 6 Months After a COVID-19 Diagnosis: A Retrospective Cohort Study

**DOI:** 10.1212/WNL.0000000000201595

**Published:** 2023-02-21

**Authors:** Maxime Taquet, Orrin Devinsky, J. Helen Cross, Paul J. Harrison, Arjune Sen

**Affiliations:** From the Department of Psychiatry (M.T., P.J.H.), University of Oxford, UK; Oxford Health NHS Foundation Trust (M.T., P.J.H.), UK; Department of Neurology (O.D.), NYU Grossman School of Medicine; UCL NIHR BRC Great Ormond Street Institute of Child Health (J.H.C.), London, UK; Young Epilepsy (J.H.C.), St Pier's Lane, Dormansland, Lingfield, UK; and Oxford Epilepsy Research Group (A.S.), NIHR Biomedical Research Centre, Nuffield Department of Clinical Neurosciences, John Radcliffe Hospital, UK.

## Abstract

**Background and Objectives:**

The relationship between COVID-19 and epilepsy is uncertain. We studied the potential association between COVID-19 and seizures or epilepsy in the 6 months after infection.

**Methods:**

We applied validated methods to an electronic health records network (TriNetX Analytics) of 81 million people. We closely matched people with COVID-19 infections to those with influenza. In each cohort, we measured the incidence and hazard ratios (HRs) of seizures and epilepsy. We stratified data by age and by whether the person was hospitalized during the acute infection. We then explored time-varying HRs to assess temporal patterns of seizure or epilepsy diagnoses.

**Results:**

We analyzed 860,934 electronic health records. After matching, this yielded 2 cohorts each of 152,754 patients. COVID-19 was associated with an increased risk of seizures and epilepsy compared with influenza. The incidence of seizures within 6 months of COVID-19 was 0.81% (95% CI 0.75–0.88; HR compared with influenza 1.55 [1.39–1.74]). The incidence of epilepsy was 0.30% (0.26–0.34; HR compared with influenza 1.87 [1.54–2.28]). The HR of epilepsy after COVID-19 compared with influenza was greater in people who had not been hospitalized and in individuals younger than 16 years. The time of peak HR after infection differed by age and hospitalization status.

**Discussion:**

The incidence of new seizures or epilepsy diagnoses in the 6 months after COVID-19 was low overall, but higher than in matched patients with influenza. This difference was more marked in people who were not hospitalized, highlighting the risk of epilepsy and seizures even in those with less severe infection. Children appear at particular risk of seizures and epilepsy after COVID-19 providing another motivation to prevent COVID-19 infection in pediatric populations. That the varying time of peak risk related to hospitalization and age may provide clues as to the underlying mechanisms of COVID-associated seizures and epilepsy.

The SARS-CoV-2 pandemic is associated with serious morbidities and mortality. By the end of April 2022, there were ∼513 million COVID-19 cases worldwide with more than 6.23 million deaths.^1^ COVID-19 infection is associated with acute neurologic manifestations, particularly encephalopathy, agitation, confusion, anosmia, ageusia, and stroke.^2,3^ Compared with influenza, people who contract COVID-19 also show an increased risk of many neurologic and psychiatric sequelae in the subsequent 6 months, with incidence highest in those admitted to an intensive care setting.^4^ COVID-19 may impair neurologic function through effects on brain endothelial cells, inflammation, cytokine storm, and other mechanisms.^5,6^

Any severe infection can cause cortical hyperexcitability through metabolic disturbances. Acute symptomatic seizures and status epilepticus are, however, rare with COVID-19.^7-9^ EEG studies in those with COVID-19 demonstrate frequent interictal epileptiform abnormalities and occasionally electrographic seizures.^10-12^ The significance of these findings and their implication for outcomes is not, though, fully understood. The incidence of acute symptomatic seizures with COVID-19 infection (∼1%) is lower than with SARS (∼2.7%) and Middle East Respiratory Syndrome (∼8.6%).^13^ Given the heterogeneous literature, it remains uncertain if COVID-19 infection predisposes patients to develop seizures or epilepsy.

Most investigations of COVID-19 and seizures have focused on the acute setting, whereas assessments of medium-term neurologic outcomes have not included epilepsy or had low case numbers.^4,14^ We, therefore, examined a large data set of healthcare records to determine the incidence of seizures and epilepsy in the 6 months after COVID-19 infection and compare these risks with matched patients after infection with influenza.

## Methods

### Data and Study Design

The study used TriNetX Analytics, a federated network of linked electronic health records recording anonymized data from 59 healthcare organizations (HCOs), primarily in the United States, totaling 81 million patients. Available data include demographics, diagnoses (ICD-10 codes), procedures (Current Procedural Terminology [CPT] codes), and measurements (e.g., blood pressure). The HCOs consist of a mixture of primary care centers, hospitals, and specialist units. They provide data from uninsured and insured individuals. Using the TriNetX user interface, cohorts are created based on inclusion and exclusion criteria, matched for confounding variables, and compared for outcomes of interest over specified periods. For further details about TriNetX, see eMethods, links.lww.com/WNL/C480.

### Standard Protocol Approvals, Registrations, and Patient Consents

Data deidentification within TriNetX is formally attested as per Section §164.514(b)(1) of the Health Insurance Portability and Accountability Act Privacy Rule, superseding TriNetX's waiver from the Western Institutional Review Board; no further ethical approval was thus needed. As we used anonymized routinely collected data, no participant consent was required.

### Cohorts

The primary cohort was defined as all patients who had a confirmed diagnosis of COVID-19 (ICD-10 code U07.1). The World Health Organization recommends using this code when COVID-19 has been confirmed by laboratory testing, irrespective of severity of clinical signs or symptoms. This was compared with a matched cohort of patients diagnosed with influenza (ICD-10 codes J09-J11) who did not have either a diagnosis of COVID-19 or a positive test for COVID-19. Cohorts included all patients who had the index event (COVID-19 or influenza) between January 20, 2020 (the date of the first recorded COVID-19 case in the United States), and May 31, 2021, and who were still alive at the end of follow-up (August 24, 2021). Individuals who had a preexisting diagnosis of epilepsy or recurrent seizures (ICD-10 G40 code) were excluded from both cohorts. More details about the cohort definition including the ICD-10/CPT codes used are provided in the eMethods, links.lww.com/WNL/C480.

### Covariates

We assessed established and suspected risk factors for COVID-19 and for more severe COVID-19 illness: age, sex, race, ethnicity, obesity, hypertension, diabetes, chronic kidney disease, asthma, chronic lower respiratory diseases, nicotine dependence, substance misuse, previous psychiatric illness, ischemic heart disease and other forms of heart diseases, socioeconomic deprivation, cancer (and hematologic cancer in particular), chronic liver disease, stroke, dementia, organ transplant, rheumatoid arthritis, lupus, psoriasis, and disorders involving an immune mechanism. To capture these risk factors in patients' health records, 58 variables were used. More details including ICD-10 codes are presented in the eMethods, links.lww.com/WNL/C480. Cohorts were matched for all these variables, as described below.

### Outcomes

The primary outcome was the 6-month incidence of the composite endpoint of epilepsy (ICD-10 code G40) or seizures (ICD-10 code R56). Secondary outcomes included either code separately.

### Statistical Analyses

Propensity score matching (performed within the TriNetX network) created cohorts with matched baseline characteristics.^15^ Propensity score 1:1 matching used a greedy nearest neighbor approach with a caliper distance of 0.1 pooled SDs of the logit of the propensity score. Any characteristic with a standardized mean difference between cohorts lower than 0.1 is considered well matched.^16^ The Kaplan-Meier estimator was used to estimate the incidence of each outcome. Hazard ratios (HRs) with 95% CIs were calculated using the Cox model, and the null hypothesis of no difference between cohorts was tested using log-rank tests. The proportional hazard assumption was tested using the generalized Schoenfeld approach. If the assumption was violated, a time-varying HR was estimated using natural cubic splines fitted to the log-cumulative hazard.^17^

Further details are in the eMethods, links.lww.com/WNL/C480. Statistical analyses were conducted in R version 3.6.3 except for the log-rank tests which were performed within TriNetX. Statistical significance was set at 2-sided *p* values <0.05. A Reporting of studies Conducted using Observational Routinely collected health Data (RECORD) statement was completed.

### Secondary Analyses

To analyze the influence of age on the results, we repeated the primary analysis in pediatric (≤16 years old) and adult (>16 years old) populations. To explore whether, and how, associations between COVID-19 and epilepsy or seizures are affected by the severity of the acute infection, we repeated the analysis separately in those who were hospitalized and those not hospitalized within 14 days of their COVID-19 or influenza diagnosis. A moderation analysis by age group (≤ vs > 16 years old) and hospitalization status was also conducted (see eMethods, links.lww.com/WNL/C480).

### Data Availability

The TriNetX system returned the results of these analyses as csv files which were downloaded and archived. Data presented in this article and the Supplement are freely accessed at osf.io/m8ht2.

## Results

The baseline demographic data of the cohorts, before and after matching, are presented in Table 1 (and eTable 1, links.lww.com/WNL/C480). Before matching, the COVID-19 data set consisted of 681,283 individuals with a mean age that was higher than the influenza data set that contained 179,561 people. There were more female patients in both groups, and this was maintained after matching. Although most of the COVID-19 and influenza cohorts were White, there was good representation of people of Black/African American and Hispanic heritage. To reduce confounders, groups were then closely matched for demographic characteristics and multiple systemic and psychiatric comorbidities, leading to matched cohorts of individuals diagnosed with COVID-19 and influenza each consisting of 152,754 individuals.

**Table 1:**
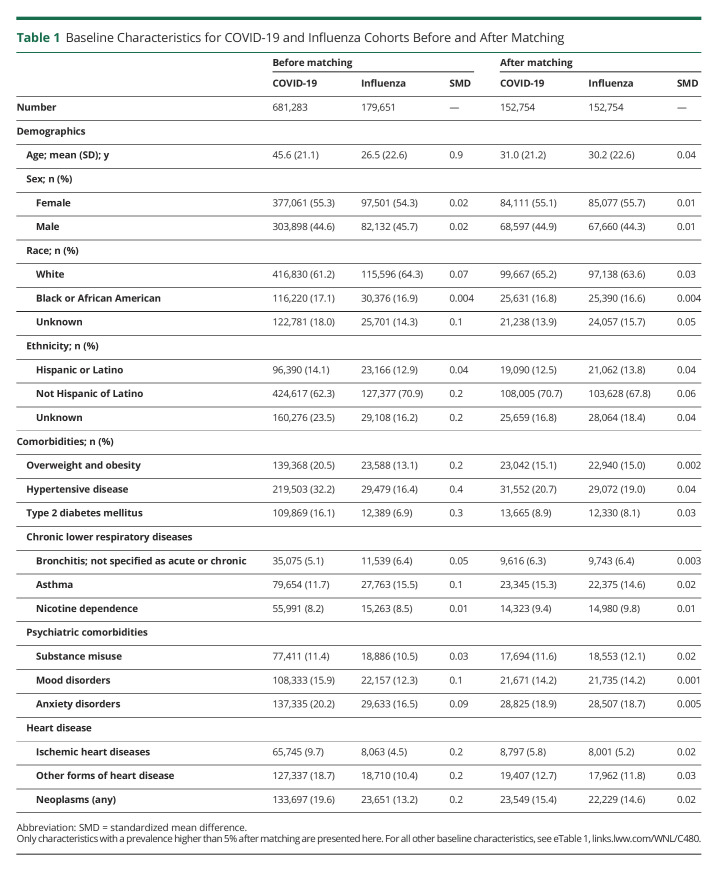
Baseline Characteristics for COVID-19 and Influenza Cohorts Before and After Matching

### Seizures and Epilepsy After COVID-19: Incidence and HRs Compared With Influenza

**Figure 1:**
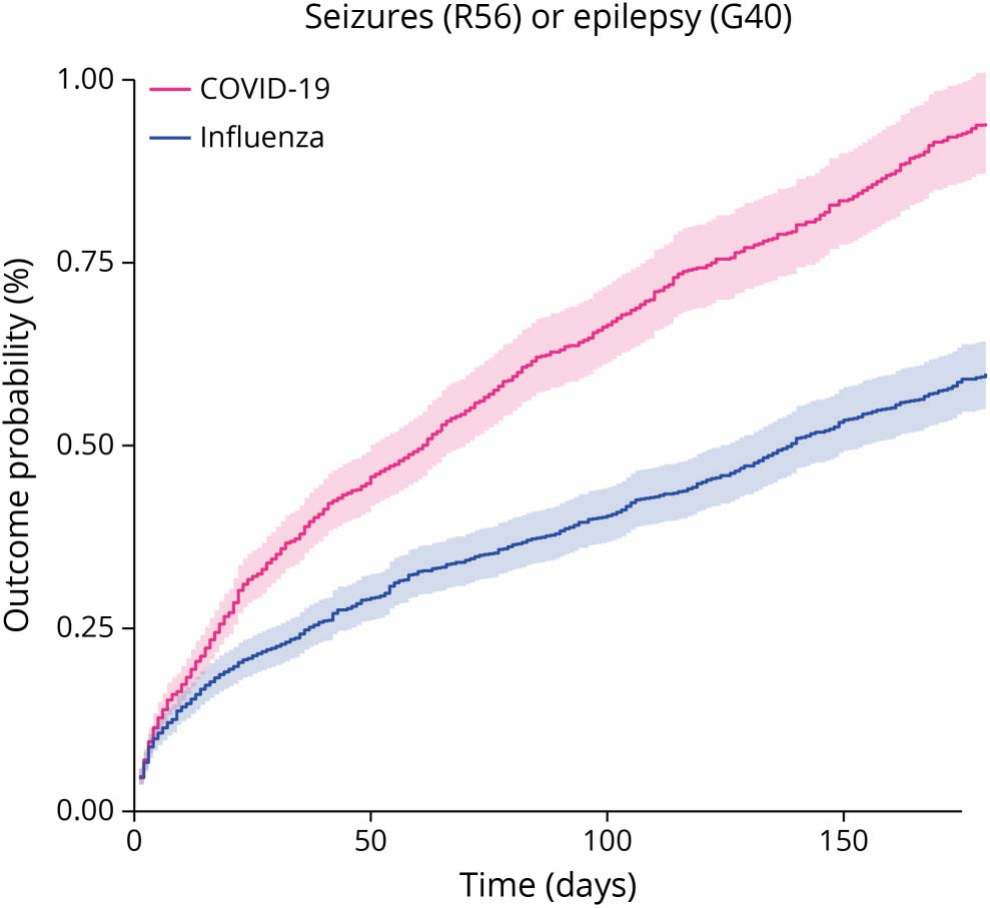
Kaplan-Meier Curves Comparing the 6-Month Cumulative Incidence of the Primary Outcome Between Matched Cohorts of Patients With COVID-19 vs Influenza An increased probability of being diagnosed with seizures or epilepsy is observed in the 6 months after COVID-19 compared with after influenza. The shaded areas around the curves represent 95% CI.

There was an increased incidence of the composite endpoint of seizures or epilepsy in the COVID-19 cohort compared with the influenza cohort (6-month cumulative incidence 0.94% vs 0.60%, HR 1.55, 95% CI 1.40–1.72, *p* < 0.0001; Figure 1; Table 2). Separately, there was an increased risk of seizures (0.81% vs 0.51%, HR 1.55, 95% CI 1.39–1.74, *p* < 0.0001) and epilepsy (0.30% vs 0.17%, HR 1.87, 95% CI 1.54–2.28, *p* < 0.0001). These findings indicate that COVID-19 infection is associated with a higher risk of both epilepsy and seizures compared with influenza.

**Table 2:**
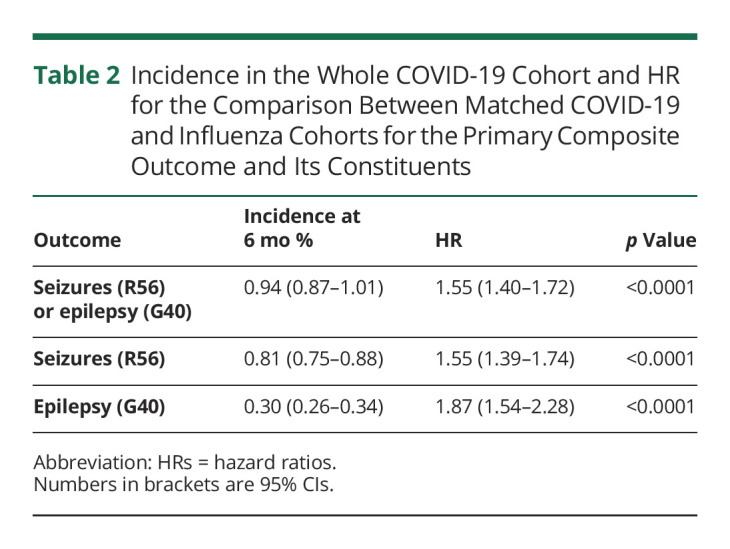
Incidence in the Whole COVID-19 Cohort and HR for the Comparison Between Matched COVID-19 and Influenza Cohorts for the Primary Composite Outcome and Its Constituents

### Secondary Analyses

#### Is the Increased Risk of Seizures and Epilepsy Age Dependent?

**Figure 2:**
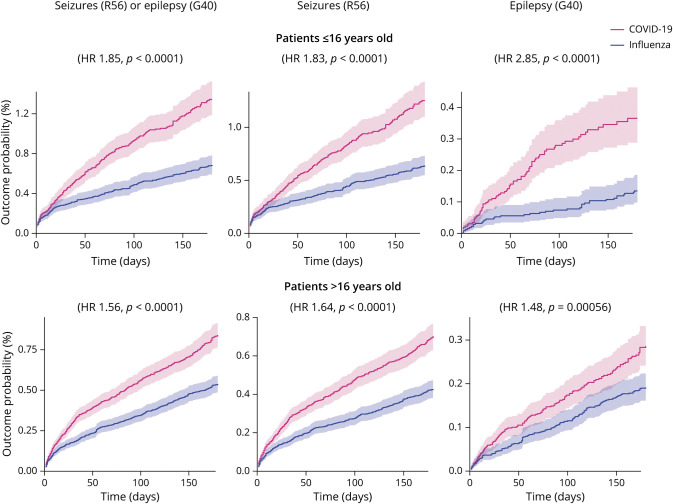
Kaplan-Meier Curves Comparing the 6-Month Cumulative Incidence of the Different Outcomes Between Matched Subgroups of Patients With COVID-19 vs Influenza Compared with influenza, COVID-19 associates with an increased probability of being diagnosed with seizures and/or epilepsy in both age groups. The risk of epilepsy was more marked in individuals younger than 16 years. The shaded areas around the curves represent 95% CI.

The results for the analysis stratified by age between children (aged ≤16 years, n = 43,231 after matching; see eTable 2, links.lww.com/WNL/C480 for baseline characteristics) and adults (aged >16 years, n = 108,116 after matching; eTable 3, links.lww.com/WNL/C480) are summarized in Figure 2 and Table 3. Compared with influenza, there was an increased risk of the composite endpoint of seizures or epilepsy after COVID-19 in both children (1.34% vs 0.69%, HR 1.85, 95% CI 1.54–2.22, *p* < 0.0001) and adults (0.84% vs 0.54%, HR 1.56, 95% CI 1.37–1.77, *p* < 0.0001). Although the contrast between COVID-19 and influenza seems more marked among children (Figure 2), there was no significant moderation by age of this composite endpoint (moderation coefficient 0.20, 95% CI −0.025 to 0.42, *p* = 0.082). There was a significantly increased risk for both seizures and epilepsy measured individually in both age groups (Figure 2). The risk of epilepsy after COVID-19 vs influenza was significantly moderated by age and more marked among children than adults (moderation coefficient 0.68, 95% CI 0.23–1.13, *p* = 0.0031).

**Table 3:**
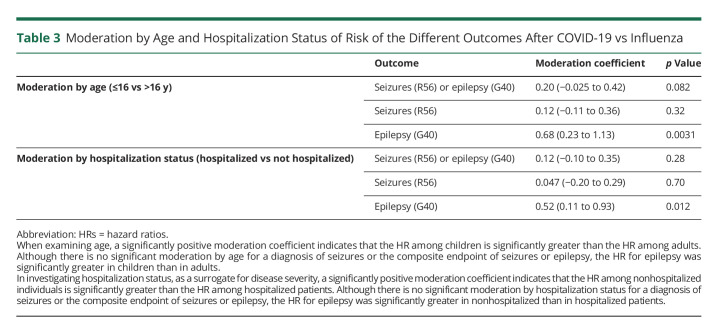
Moderation by Age and Hospitalization Status of Risk of the Different Outcomes After COVID-19 vs Influenza

#### Is the Increased Risk of Seizures and Epilepsy Dependent on the Severity of COVID-19 Infection as Proxied by Hospitalization?

**Figure 3:**
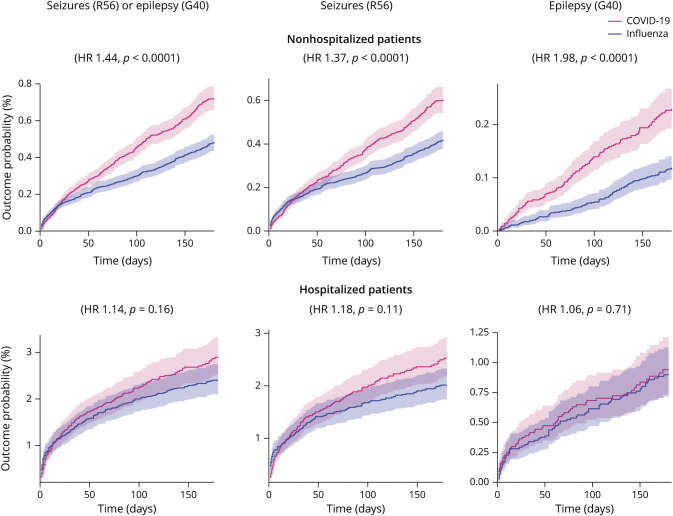
Kaplan-Meier Curves Comparing the 6-Month Cumulative Incidence of the Primary Outcome Between Matched Subgroups of Nonhospitalized and Hospitalized Patients With COVID-19 vs Influenza In people who were hospitalized the risks of seizures and/or epilepsy were similar after COVID-19 and influenza infections. In nonhospitalized patients, COVID-19 associated with significantly increased risks of seizures and/or epilepsy. The shaded areas around the curves represent 95% CI.

The results for the analysis stratified by hospitalization status, between nonhospitalized (n = 139,490 after matching; see eTable 4, links.lww.com/WNL/C480 for baseline characteristics) and hospitalized individuals (n = 11,090 after matching; see eTable 5, links.lww.com/WNL/C480) are summarized in Figure 3 and Table 3. Compared with influenza, there was a significantly increased risk of the composite endpoint of seizures or epilepsy after COVID-19 in nonhospitalized individuals (0.72% vs 0.48%, HR 1.44, 95% CI 1.27–1.63, *p* < 0.0001) but not in hospitalized individuals (2.90% vs 2.40%, HR 1.14, 95% CI 0.95–1.38, *p* = 0.16). However, hospitalization status was not a significant moderator (moderation coefficient 0.12, 95% CI −0.10 to 0.35, *p* = 0.28). Similarly, there were significantly increased risks in both seizures and epilepsy measured individually in the nonhospitalized group only (Figure 3). Hospitalization status was a significant moderator for the association between COVID-19 and epilepsy (with the association being more marked among nonhospitalized patients; moderation coefficient 0.52, 95% CI 0.11–0.93, *p* = 0.012), but not for seizures (moderation coefficient 0.047, 95% CI −0.20 to 0.29, *p* = 0.70).

#### When Is the Peak Risk of “Seizures or Epilepsy” After COVID-19 Compared With Influenza?

**Figure 4:**
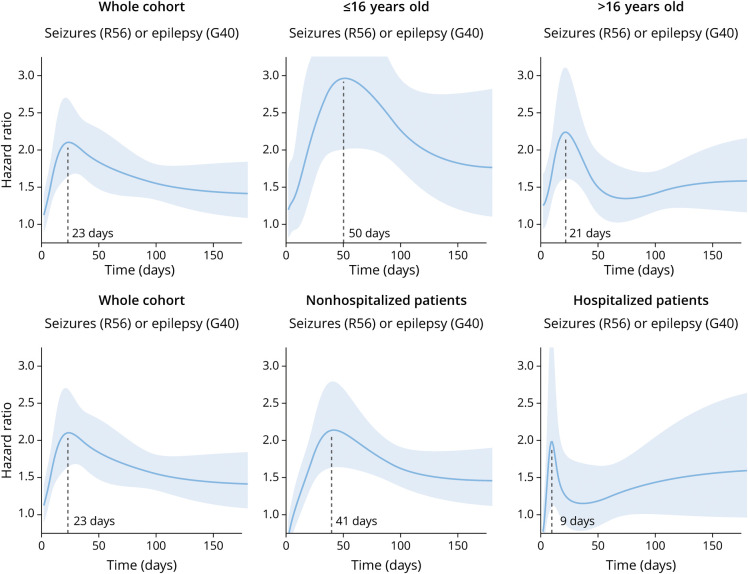
Time-Varying Hazard Ratios for the Primary Analysis (Left) and Nonhospitalized/Hospitalized and Pediatric/Adult Subgroups The time of the peak HR is noted on the x-axis. The left-most panel in each row is identical to facilitate comparison. The peak HR in the whole cohort is at 23 days, similar to that seen in those older than 16 years. In those younger than 16 years, the peak is delayed to 50 days and, at that point, the HR is nearly 3.0. Hospitalized patients show a peak HR at 9 days, while in nonhospitalized patients, the peak HR is at 41 days.

We performed a post hoc analysis of time-varying HRs for the composited endpoint of seizures or epilepsy across the whole cohort (Figure 4) and separately according to hospitalization status, and in the 2 age groups. Across the whole cohort, the peak time for the HR of seizures or epilepsy between COVID-19 and influenza was 23 days after infection. The peak time for the HR was 21 days in adults and 50 days in children. At 50 days of postinfection, children were almost 3 times more likely to have seizures or epilepsy diagnosed after COVID-19 infection than after influenza. Among individuals hospitalized with COVID-19 or influenza, the HR for seizures or epilepsy peaked at 9 vs 41 days in those who were not hospitalized. At that timepoint, nonhospitalized people were more than twice as likely to have seizures or epilepsy diagnosed post–COVID-19 compared with influenza.

## Discussion

In a large electronic health records network, our study revealed that COVID-19 is associated with an increased risk of seizures or epilepsy when compared with matched patients with influenza over 6-month time horizon from the date of infection. Although the risk of epilepsy or seizures was significantly raised after COVID-19 compared with influenza, the absolute risk remains low (affecting less than 1% of all patients with COVID-19), consistent with other studies.^13,18,19^ The relative risk of epilepsy or seizures after COVID-19 infection, compared with after being infected with influenza, was more marked among children and nonhospitalized individuals over the 6-month time horizon.

The elevated risk among children was unexpected, although it is appreciated that COVID-19 affects adults and children differently.^20-23^ Pulmonary disease is the main manifestation in adults, while immune-mediated inflammatory response with or without multisystem inflammatory syndrome in children was the major manifestations of COVID-19 in children. Children with neurologic manifestations can be more likely to have positive COVID-19 antibodies either alone or in combination with COVID-19 PCR positivity. Those without neurologic manifestations often only had positive COVID-19 PCR results, suggestive of acute infection.^20^

Many immune-mediated parainfectious CNS illnesses manifest sometime after the offending viral infection,^24^ consistent with the delayed peak in the risk of epilepsy in our COVID-19 pediatric cohort. Immune-mediated or inflammatory-mediated mechanisms of COVID-19 could contribute to epileptogenesis in the developing brain or unmask a previous predisposition to seizures. Epilepsy has neurodevelopmental, psychological, social, and educational consequences.^25,26^ Although the infection is often mild in children, neurologic consequences of COVID-19 may potentially be more severe.^27^ Our data provide additional support for preventing COVID-19 infection in children, which can inform the risks-benefits balance of vaccination in pediatric populations.

A similar immune-mediated mechanism might account for the differences seen in nonhospitalized patients. In this group, there was a higher risk of seizures or epilepsy after COVID-19 compared with influenza, and this relative risk gradually increased over time, peaking at around 6 weeks after the acute infection. An increasing HR over time only implies that the incidence in 1 group increases *relative* to the other group. Cautious interpretation is therefore warranted. The observation of an increasing risk of seizures or epilepsy over a few weeks post–COVID-19 is, though, potentially consistent with an immune-mediated etiology. There should be greater attention to those presenting with subtle features of seizures, for example, focal aware seizures, particularly in the 3 months after less severe COVID-19 infection. By contrast, severe infections can directly lower seizure threshold owing to metabolic disturbances, fever, sleep deprivation, and other factors. This is consistent with our observation that the risk of epilepsy or seizure in hospitalized patients with COVID-19 peaks shortly after infection, while not being significantly greater than in hospitalized patients with influenza over the whole 6-month follow-up period.

Although these data offer insights into whether COVID-19 may contribute to seizures and epileptogenesis, much remains unanswered. We sought to determine whether an underlying cause of seizures could be identified, particularly considering if stroke, a potential consequence of COVID-19,^28-30^ may be the main cause of COVID-19–related seizures or epilepsy. The data did not allow this to be answered because of the limited number of patients with a sequential diagnosis of COVID-19, stroke, and subsequent epilepsy or seizures. Since most people who experienced a stroke were likely hospitalized,^29^ and that the increased risk of seizures or epilepsy was mainly seen in nonhospitalized patients, it is perhaps less likely that stroke was a major factor in the development of epilepsy.

The long-term outcomes of patients diagnosed with seizures post–COVID-19 remain poorly characterized. People and clinicians may choose not to initiate medication, even after 2 unprovoked seizures, if these occur proximal to COVID-19 infection and perhaps particularly if EEG and MRI do not suggest an underlying substrate for seizures. It will be important to monitor these individuals to determine whether further seizures supervene. In those who do start medication, especially children, it will be crucial to track seizure profiles and long-term neurodevelopmental/neurocognitive outcomes.

Our study shows that the absolute risk of epilepsy and seizures after COVID-19 infection is comparatively low. The relative risk is, though, greater after COVID-19 infection than after influenza, particularly in people who were not hospitalized and in children (aged less than 16 years). The peak HR in these more susceptible groups occurred some weeks after infection with COVID-19, potentially suggesting an immune-mediated etiology. Other study designs are required to further investigate possible underlying mechanisms.

As seizures and epilepsy remain relatively rare outcomes after COVID-19, we support continued pooling of data across multiple centers and establishing long-term open access repositories for the reporting of post–COVID-19 seizures and epilepsy. Transparent reporting of outcomes is crucial to better understanding how COVID-19 may interrelate with seizure disorders.

This study has several limitations beyond those inherent to research using electronic health records^4,31^ (summarized in the eMethods, links.lww.com/WNL/C480), such as the unknown completeness of records, no validation of diagnoses, and sparse information on socioeconomic and lifestyle factors. We cannot comment on people who were infected with COVID-19 but could not be matched to those from our influenza cohort. We do not know with which SARS-CoV-2 variant individual patients were infected, nor whether they had previously been vaccinated against SARS-CoV-2, and this might influence the likelihood of developing seizures. As the study is entirely reliant on people being coded as having COVID-19 to enter the data set, this study cannot comment on outcomes in patients infected with SARS-CoV-2 but who were not tested or diagnosed with COVID-19.

We matched a large number of people who had influenza to COVID-19 cases. The comparison cohort was selected to be contemporaneous to the COVID-19 cohort to limit the effect of contextual factors (e.g., strained health services) on outcomes. The incidence of influenza has decreased during the COVID-19 pandemic, so those affected might not be representative of people diagnosed with influenza before the pandemic. Very similar HRs were, though, observed for other neurologic outcomes when comparison was made with cohorts of patients diagnosed with influenza in 2018 and 2019.^4^ Conversely, we did not compare the risk of epilepsy and seizures between a COVID-19 cohort and the general population, and it is possible that the corresponding HR would be greater than those observed when comparing COVID-19 with influenza. In addition, we cannot compare post–COVID-19 sequelae with infections with more “epileptogenic” viruses, such as herpes simplex virus,^32^ because there are insufficient case numbers.

There are intrinsic difficulties when coding for epilepsy and seizures. COVID-19 associates with psychological comorbidity, both in those with preexisting seizures^33-35^ and in those who do not have epilepsy.^4^ Although psychological stresses can contribute to the development of epilepsy, this can also precipitate psychological nonepileptic attacks (PNES, dissociative seizures, and functional seizures).^36^ PNES may be miscategorized as seizures or epilepsy, and this may be overrepresented in the COVID-19 cohort. Seizures are also a nuanced, clinical diagnosis, and it is possible that, for example, cardiovascular episodes of collapse or metabolic derangement (for example, hypoglycaemia) may be coded as “seizure” or even “epilepsy.” Similar limitations do, though, also apply to those infected with either COVID-19 or influenza helping to validate the approach presented here.

## Glossary

CPT: Current Procedural Terminology
HRs: hazard ratios
HCOs: healthcare organizations
ICD: *International Classification of Diseases*
PNES: psychological nonepileptic attacks
